# A Study on Serum Antithyroglobulin Antibodies Interference in Thyroglobulin Measurement in Fine-Needle Aspiration for Diagnosing Lymph Node Metastasis in Postoperative Patients

**DOI:** 10.1371/journal.pone.0131096

**Published:** 2015-06-29

**Authors:** Hyun Joo Shin, Hye Sun Lee, Eun-Kyung Kim, Hee Jung Moon, Ji Hye Lee, Jin Young Kwak

**Affiliations:** 1 Department of Radiology, Severance Hospital, Research Institute of Radiological Science, Yonsei University, College of Medicine, Seoul, Korea; 2 Biostatistics Collaboration Unit, Medical Research Center, Yonsei University, College of Medicine, Seoul, Korea; University of Toronto, CANADA

## Abstract

**Purpose:**

Thyroglobulin measurement in fine-needle aspiration washout fluid (FNA-Tg) is widely used for detection of lymph node metastasis (LNM) in patients with papillary thyroid cancer (PTC). Recent studies suggested that serum anti-thyroglobulin antibodies (TgAbs) could interfere with FNA-Tg. We evaluated whether TgAbs can affect FNA-Tg when diagnosing LNM in postoperative patients with PTC.

**Methods:**

From November 2006 to June 2011, a total of 239 LNs from 201 patients who underwent bilateral thyroidectomy and radioactive iodine ablation therapy were included. The interactions between FNA-Tgs and serum TgAbs, and diagnostic performances between FNA with additional FNA-Tg and FNA alone according to the presence of serum TgAbs were evaluated using the generalized linear mixed model and the bootstrap method.

**Results:**

From 106 (44.4%) malignant and 133 (55.6%) benign LNs, there were 32 (13.4%) LNs with detectable serum TgAb levels and 207 (86.6%) LNs with undetectable serum TgAb levels. In logistic regression analysis, a significant negative interaction was observed between FNA-Tgs and serum TgAbs (p = 0.031). In the absence of serum TgAbs, the diagnostic performances were superior in the FNA with FNA-Tg than in the FNA only. However, in the presence of serum TgAbs, the diagnostic performances of the FNA with FNA-Tg were not significantly different from the FNA only, even with a different cutoff value of FNA-Tg.

**Conclusions:**

Serum TgAbs may interfere with FNA-Tg studies and caution is advised while analyzing FNA-Tg for detection of LNM in patients with PTC.

## Introduction

Papillary thyroid carcinoma (PTC) usually has a good prognosis with indolent course. However, loco-regional recurrence is not negligible, ranging from 5–20% in patients who undergo surgery for PTC [1, 2]. The most frequently involved site of loco-regional metastasis of PTC is the neck, and the incidence of lymph node metastasis (LNM) is relatively high with values reported from 3.1% to 28.9%, which can influence the prognosis [3–5]. Therefore, several methods are generally used to detect metastases or recurrences of PTC, such as diagnostic whole body scan, neck ultrasonography (US) and serum thyroglobulin (Tg) measurements [6–10].

To diagnose loco-regional lymph node metastases, ultrasound-guided fine-needle aspiration (US-FNA) is essential, but 5–10% of nondiagnostic results and 6–8% of false-negative results have also been found with the procedure [11, 12]. Therefore, an additional test of Tg measurements in FNA washout fluid (FNA-Tg) is widely used with a high sensitivity (88.3–96.1%) and specificity (81.6–96%) for detection of early LNM in patients with PTC after thyroid surgery as well as before thyroid surgery [12–19]. Serum anti-Tg antibodies (TgAbs) are present in approximately 10% of the general population and 25% of thyroid cancer patients [10, 20]. Although it is already well known that serum TgAbs can falsely lower serum Tg measurements, serum TgAbs have not been thought to influence the detection of FNA-Tgs [7, 10]. Recently, however, it has been suggested in some studies that high serum TgAbs above 20 and 60 IU/mL can interfere with FNA-Tg measurements [21, 22]. Therefore, the aim of our study was to evaluate whether serum TgAbs can affect FNA-Tg detection for diagnosing LNM in postoperative patients with papillary thyroid cancer.

## Materials and Methods

The institutional review board of Severance hospital approved of this retrospective observational study and required neither patient approval nor informed consent for our review of patients’ images and patients’ medical records. However, written informed consent was obtained from all patients for US-FNAs prior to each procedure as part of our hospital’s daily practice. Our institutional review board waived the need for written informed consent from the patients. Patients' records and information were anonymized and de-identified prior to analysis.

### Patients

From November 2006 to June 2011, 210 consecutive patients underwent FNA and FNA-Tg measurements to detect lymph node recurrences of PTC at our institution (a referral center). All patients underwent bilateral thyroidectomy and remnant ablation with radioactive iodine [131-iodine (^131^I)]. Eight patients were excluded because they did not undergo surgical excision or long-term imaging follow-up for at least 1 year. One patient was also excluded because she had another malignancy. Of the 239 LNs in 201 patients, 170 patients had one LN, 24 had two LNs and 7 had 3 LNs. Of the 201 patients, 8 patients had both malignant and benign LNs (6 patients had one malignant and one benign LN, one patient had two malignant and one benign LNs, and one patient had one malignant and two benign LNs). One hundred twelve patients had benign LNs only (102 patients had one benign LN, 8 patients had two, and 2 patients had three benign LNs), and 81 patients had malignant LNs only (68 patients had one malignant LN, 10 patients had two, and 3 patients had three malignant LNs). Patients ranged in age from 20 to 83 years (mean, 48.3 years).

### Ultrasound-guided fine needle aspiration with Tg test

US-FNAs were performed by one of 21 radiologists who were aware of the patients’ clinical history. US-FNAs were performed with a 23-gauge needle attached to a 2-mL disposable plastic syringe. Each cervical lymph node was aspirated at least twice. The materials obtained from FNA were smeared on glass slides for conventional cytology. All smears were placed in 95% alcohol for Papanicolaou staining and the remaining aspirates in the syringe and needle were rinsed with normal saline and 1 mL of rinsed washout was submitted for Tg measurement (FNA-Tg) [12, 16]. All patients were taking levothyroxine for TSH suppression at the time of FNA-Tg measurement.

### Biochemical analysis

Serum Tg and FNA-Tg were assayed with a monoclonal antibody immunoradiometricassay (IRMA; CIS Bio International, Gif-sur-Yvette, France). Analytical sensitivity, defined as the smallest detectable concentration different from zero with a probability of 95%, was 0.2 ng/ml. Functional sensitivity, calculated with the imprecision profile for a coefficient of variation equal to 20%, was 0.7 ng/ml [12, 14, 16, 17, 23]. Serum TgAbs were measured by radioimmunoassay (Brahms, Hennigsdorf/Berlin, Germany) with a functional sensitivity of 10 IU/mL: antibody positivity was defined as a TgAb value exceeding 60 U/mL [24]. Serum TSH levels were measured by a radioimmunoassay (Trinity Biotech, Co. Wicklow, Ireland) with a reference range of 0.4–3.1 μIU/mL and a functional sensitivity of 0.0038 mIU/L.

### Cytology and pathology

Positive cytology was defined as a metastasis from thyroid carcinoma or foamy macrophages without malignant cells in the cytology report [23, 25–27]. A previous study showed that metastasis on a pathologic report could be proven not only with a previous cytology result of metastasis, but also with foamy macrophages without malignant cells [23]. Negative cytology included benign cytology such as reactive hyperplasia, and other benign lymphadenitis. In addition, we included the non-diagnostic cytology of paucicellular cytology from insufficient material to the negative cytology category because cytology results revealed no definite malignant cells.

Positive diagnoses were made based on histological or cytological findings. Negative diagnoses were based on no evidence of malignant LN on histological findings or absence of evolution or decrease on US without cytological evidence of malignancy at 1 year or more of follow-up [23, 28].

### Data and Statistical Analysis

Patients with detectable serum TgAbs (sTgAb^+^) and undetectable serum TgAbs (sTgAb^-^) were compared according to patient characteristics. Categorical variables were summarized by frequencies and percentages. Continuous variables were presented as means ± standard deviation (SD) or as medians with interquartile range. We used the χ^2^ test to determine differences between metastatic and benign lymph nodes according to categorical variables. For those values not normally distributed, a nonparametric method such as the Mann-Whitney *U* test was used. The independent two-sample t-test was used to compare patient characteristics between the two groups.

Values with a skewed distribution were logarithmically transformed and analyzed. Normality of the variables was tested using the Kolmogorov-Smirnov test. Values of FNA-Tgs were logarithmically transformed before analyses to adjust for skewed distributions. To examine whether the associations between FNA-Tgs and metastases were modified by serum TgAbs, we tested their interactions on a multiplicative scale. Specifically, the interaction term for FNA-Tg x TgAb was included in the generalized linear mixed model for binary data, considering the correlation between the LNs of the same patients.

Due to the interaction between FNA-Tgs and serum TgAbs, we divided cases into the sTgAb^+^ group and sTgAb^-^ group. Receiver operating characteristic (ROC) analyses were performed to determine the cutoff values of FNA-Tg to diagnose malignant LNs in the two groups, respectively. Youden’s method was used to find an optimal cutoff point in the ROC curve to maximize sensitivity and specificity. The area under the ROC curves (AUCs) of the groups were also obtained with confidence intervals. The diagnostic performances of FNA-Tg at an optimal cutoff point in the ROC curves were assessed by calculating sensitivity, specificity, accuracy, positive predictive value (PPV) and negative predictive value (NPV) for each group separately. We compared the diagnostic performances of FNA-Tg according to the presence of serum TgAbs and also compared diagnostic performances between the FNA only group and the FNA with additional FNA-Tg group using the generalized linear mixed model for binary data. To compare AUCs, the bootstrap method was used with resampling done 1000 times. P values were computed on the basis of 1000 bootstrap samples.

For all analyses, results were considered statistically significant if the *p* value was < 0.05. For statistical analysis, we used a computerized statistics program (SAS version 9.2 for Windows; SAS Institute, Cary, NC).

## Results

In this study, 239 LNs in 201 patients were included. There were 106 (44.4%) malignant LNs and 133 (55.6%) benign LNs. From those 106 LNs which were classified as malignant in our study, 3 LNs had cytology results of ‘paucicellular smear showing several scattered hemosiderin-laden macrophages’. However, the final pathology after excision of suspected LNs turned out to be metastatic carcinomas from papillary thyroid carcinoma in these three LNs. S1 Material showed the part of detailed information of the patients including their FNA-Tg and TgAb levels. Table 1 showed the clinical characteristics and US features of the LNs without and with metastasis. On univariate analysis, LNMs were significantly associated with male gender, larger primary tumor size, presence of lymph node metastasis at the initial operation, and higher serum Tg levels (Table 1).

**Table 1:** Patient Characteristics according to Final Diagnosis.

Characteristics	Malignancy	Benign	*P* value
Number of patients, n	86	115	
Number of LNs, n	106	133	
Gender, male/female (%)	38/68 (35.8)	28/105 (21.1)	0.011^b^
Mean age, years	47.4±14.4 (20–83)	49.1±12.9 (20–78)	0.340 ^c^
Primary tumor ^a^			
Size, mm (range)	18.6 ± 12.2 (3–24)	14.3 ±11.0 (3–31)	0.020 ^c^
Multiplicity, yes/no (%)	23/42 (35.4)	34/62 (35.4)	0.997^b^
LN metastasis, yes/no (%)	54/11 (83.1)	62/34 (64.6)	0.010^b^
Extrathyroid extension, yes/no (%)	45/20 (69.2)	64/32 (66.7)	0.733^b^
Serum Tg, ng/ml (interquartile range)	0.7 (3.3)	0.2 (0.1)	< 0.001^d^
Serum Tg antibody, yes/no (%)	16/90 (15.1)	16/117 (12.0)	0.489^b^

Results are expressed as number (percent), mean ± standard deviation (range) or median (interquartile range)

^a^Evaluation of the characteristics of the primary tumor was possible for 123 LNs in 121 patients who had available initial operation data.

^b^Derived from the Chi-square test.

^c^Derived from the Student’s *t* test.

^d^Derived from the Mann-Whitney *U* test.

LN, lymph node; Tg, thyroglobulin.

There were 32 (13.4%) LNs in the sTgAb^+^ group and 207 (86.6%) LNs in the sTgAb^-^ group. The value of the FNA-Tg level in metastatic LNs was 347.9 ± 189.9 ng/mL (243.9 ± 224.2 ng/mL in the sTgAb^+^ group, 366.3 ± 178.2 ng/mL in the sTgAb^-^ group). The value of the FNA-Tg level in benign LNs was 1.2 ± 5.1 ng/mL (5.7 ± 13.9 ng/mL in the sTgAb^+^ group, 0.5 ± 1.2 ng/mL in the sTgAb^-^ group). For diagnosing LNM, there were significant interactions between FNA-Tgs and serum TgAbs (*p* = 0.022 without adjusting lymph node size, *p* = 0.031 with adjusting size) in logistic regression analyses. The odd ratios (OR) for the prediction of LNMs using FNA-Tgs were decreased from 2.685–2.780 in the sTgAb^-^ group to 1.707–1.779 in the sTgAb^+^ group. In the subgroup analysis, the ORs of FNA-Tg were 2.685 for the prediction of LNMs in the sTgAb^-^ group (95% CI 2.102–3.428, *p* < 0.001) and 1.707 in the sTgAb^+^ group (95% CI 1.105–2.637, *p* = 0.027) on univariate analysis. On multivariate analysis, the ORs of FNA-Tg were 2.780 in the sTgAb^-^ group (95% CI 2.130–3.629, *p* < 0.001), and 1.779 in the sTgAb^+^ group (95% CI 1.013–3.124, *p* = 0.047).

**Fig 1:**
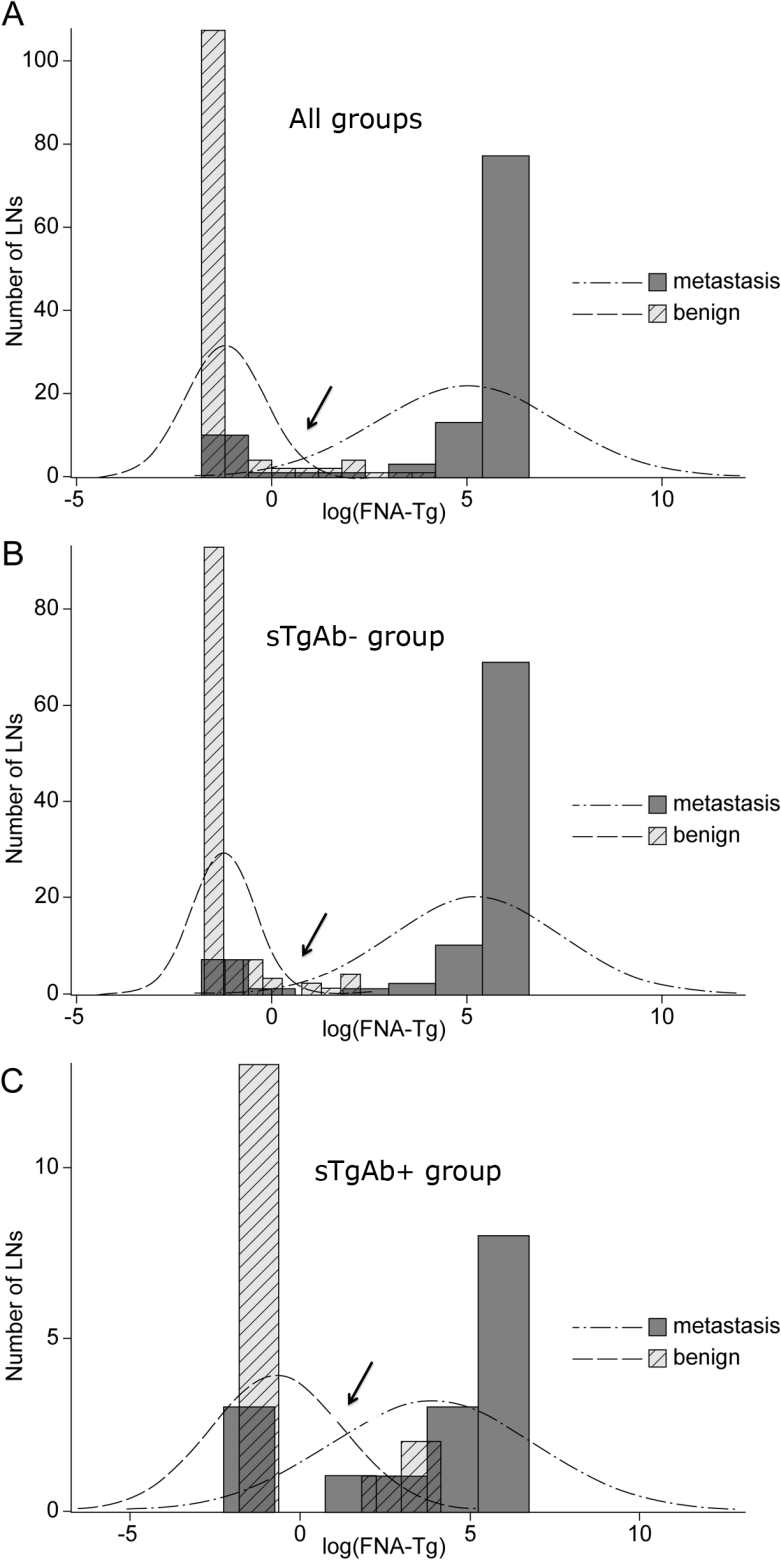
The distribution of FNA-Tg in lymph node metastasis and benign groups according to the TgAb. (A) Comparison of FNA-Tg levels in all groups. (B) Comparison of FNA-Tg levels in the sTgAb^-^ group. (C) Comparison of FNA-Tg levels in the sTgAb^+^ group. The cutoff values of FNA-Tg between malignant and benign lymph nodes were presented as arrows (19.9 [= exp (2.989)] ng/mL in a, 19.9 [= exp (2.989)] ng/mL in b, 38.3 [= exp (4.256)] ng/mL in c, respectively). The abscissa represents the logarithmically transformed values of FNA-Tgs to adjust for skewed distributions. sTgAb-, undetectable serum TgAbs; sTgAb+, detectable serum TgAbs; All groups, sTgAb^-^ and sTgAb^+^ groups being included together; FNA-Tg, thyroglobulin level in FNA washout fluid; LN, lymph node.

The distribution of FNA-Tg levels was shown according to the presence of serum TgAbs (Fig 1). The cutoff values of FNA-Tg for the prediction of LNMs were 19.9 [= exp (2.989)] ng/mL in the all and sTgAb^-^ group and 38.3 [= exp (4.256)] ng/mL in the sTgAb^+^ group. Of the 32 LNs in the sTgAb^+^ group, 4 out of 16 metastatic LNs (25%) had FNA-Tg levels less than 38.3 ng/mL. Furthermore, of the 207 LNs in the sTgAb- group, 8 out of 90 metastatic LNs (8.9%) had FNA-Tg levels less than 19.9 ng/mL. In addition, of the 32 LNs in the sTgAb+ group, 1 out of 16 benign LNs (6.3%) had a FNA-Tg level higher than 38.3 ng/mL, and of the 207 LNs in the sTgAb- group, no LNs from the 117 benign LNs (0%) showed a FNA-Tg level higher than 19.9 ng/mL in our study. When applying the cutoff value of 19.9 ng/mL, all diagnostic performances except sensitivity were significantly increased for diagnosing LNMs in the sTgAb^-^ group compared with those in the sTgAb^+^ group (Table 2). Sensitivity was also increased although without statistical significance. The predictive power of FNA-Tgs in the sTgAb^-^ group (AUC 0.956; 95% CI 0.924–0.983) was significantly superior to that of the sTgAb^+^ group (AUC 0.814; 95% CI 0.670–0.941) in predicting LNMs (*p* = 0.024). When a cutoff value of 38.3 ng/mL of FNA-Tgs in sTgAb^+^ was applied, specificity, PPV, NPV and diagnostic accuracy were significantly decreased in the sTgAb^+^ group with a cutoff value of 38.3 ng/mL, compared to the sTgAb^-^ group with a cutoff value of 19.9 ng/mL.

**Table 2:** Diagnostic performances of FNA-Tg according to the presence of serum TgAbs.

	sTgAb^-^ ^a^	sTgAb^+^ ^a^	sTgAb^+^ ^b^	*p* value^c^	*p* value^d^
Sensitivity, %	91.1 (85.2–97.0)	75.0 (53.8–96.2)	75.0 (53.8–96.2)	0.057	0.057
Specificity, %	100 (100–100)	87.5 (71.3–100)	93.8 (81.9–100)	<0.001	0.005
PPV, %	100 (100–100)	85.7 (67.4–100)	92.3 (77.8–100)	<0.001	0.009
NPV, %	93.6 (89.3–97.9)	77.8 (58.6–97.0)	79.0 (60.6–97.3)	0.021	0.029
Accuracy, %	96.1 (93.5–98.8)	81.3 (67.7–94.8)	83.3 (70.0–96.7)	0.001	0.006
AUC	0.956 (0.924–0.983)	0.814 (0.670–0.941)	0.844 (0.682–0.962)	0.024	0.066

Data in parentheses are 95% confidence intervals.

^a^Cutoff value of FNA-Tg was 19.9 ng/mL.

^b^Cutoff value of FNA-Tg was 38.3 ng/mL.

^c^ *p*-value between the sTgAb- and sTgAb+ groups when applying the same FNA-Tg cutoff value of 19.9 ng/mL (sTgAb^-a^ vs. sTgAb^+ b^).

^d^ *p*-value between the sTgAb- group with a FNA-Tg cutoff value of 19.9 ng/mL and the sTgAb+ group with a FNA-Tg cutoff value of 38.3 ng/ mL (sTgAb^-a^ vs. sTgAb^+ b^).

FNA-Tg, thyroglobulin level in FNA washout fluid; sTgAb-, undetectable serum TgAbs; sTgAb+, detectable serum TgAbs; PPV, positive predictive value; NPV, negative predictive value; AUC, the area under the ROC curves.

Additionally, we compared diagnostic performances between FNA alone and FNA with additional FNA-Tgs (Table 3). When we included all groups, the combination of FNA and FNA-Tgs with a cutoff value of 19.9 ng/mL showed significantly superior sensitivity and NPV to FNA cytology alone (*p* = 0.012 and 0.013, respectively). In the sTgAb^-^ group, the combination of FNA and FNA-Tgs with a cutoff value of 19.9 ng/mL showed significantly superior sensitivity, NPV, accuracy (all, *p* = 0.02) and AUC (*p* < 0.001), to FNA alone. In the sTgAb^+^ group, diagnostic performances of the combination of FNA and FNA-Tgs with a cutoff value of 38.3 ng/mL were not significantly different from those of FNA cytology alone.

**Table 3:** Diagnostic performance comparison between FNA alone and the combination of FNA with FNA-Tg.

	All (*n* = 239)			sTgAb^-^ (*n* = 207)			sTgAb^+^ (*n* = 32)		
	FNA	FNA with FNA-Tg^a^	*p* value	FNA	FNA with FNA-Tg^a^	*p* value	FNA	FNA with FNA-Tg^b^	*p* value
Sensitivity, %	91.5 (86.2–96.8)	97.2 (94.0–100)	0.012	91.1 (85.2–97.0)	96.7 (93.0–100)	0.021	93.8 (81.9–100)	100 (100–100)	0.302
Specificity, %	100 (100–100)	98.5 (96.4–100)	0.154	100 (100–100)	100 (100–100)	> 0.999	100 (100–100)	93.8 (81.9–100)	0.302
PPV, %	100 (100–100)	98.1 (95.5–100)	0.153	100 (100–100)	100 (100–100)	> 0.999	100 (100–100)	94.1 (82.9–100)	0.303
NPV, %	93.7 (89.7–97.7)	97.8 (95.3–100)	0.013	93.6 (89.3–97.9)	97.5 (94.7–100)	0.023	94.1 (82.9–100)	100 (100–100)	0.303
Accuracy, %	96.2 (93.8–98.7)	97.9 (96.1–99.7)	0.156	96.1 (93.5–98.8)	98.6 (96.9–100)	0.024	96.9 (90.9–100)	96.9 (90.9–100)	> 0.999
AUC	0.958 (0.930–0.981)	0.978 (0.958–0.995)	0.072	0.955 (0.924–0.983)	0.983 (0.961–1.000)	<0.001	0.970 (0.893–1.000)	0.967 (0.893–1.000)	0.9

Data in parentheses are 95% confidence intervals.

^a^Cutoff value of FNA-Tg was 19.9 ng/mL.

^b^Cutoff value of FNA-Tg was 38.3 ng/mL.

All, groups including sTgAb- and sTgAb+; sTgAb-, undetectable serum TgAbs; sTgAb+, detectable serum TgAbs; FNA, fine needle aspiration; FNA-Tg, thyroglobulin level in FNA washout fluid; PPV, positive predictive value; NPV, negative predictive value; AUC, the area under the ROC curves.

## Discussion

Additional FNA-Tg measurements to FNA are well known to have high sensivity, NPV and accuracy when predicting LNMs in the patients who undergo thyroidectomy or not, when compared to performing FNA alone (Table 4) [7, 8, 12, 14, 15, 18, 22]. However, there have only been a few studies about whether serum TgAbs can influence FNA-Tg detection [7, 10, 21, 22]. Baskin first investigated the role of FNA-Tgs in detecting recurrent PTCs at suspicious lymph nodes in 21 patients who had undergone a total or near-total thyroidectomy followed by ^131^I ablation [10]. In that study, serum TgAbs did not interfere with FNA-Tg measurements when diagnosing recurrences in the neck and he speculated that intracellular Tg might not be exposed to circulating TgAbs [10]. Then, Boi et al. evaluated the usefulness of FNA-Tg in relation to the presence of serum TgAbs for LNM diagnosis in 73 patients with differentiated thyroid carcinoma before and after thyroidectomy [7]. Serum TgAbs were detected in FNA washout fluids in at least 25% of patients and Boi et al. suggested the possibility of contamination of blood or FNA washout fluid analysis by serum TgAbs [7]. However, they concluded that serum TgAbs did not lower the sensitivity and specificity of FNA-Tg measurements, because the FNA-Tg levels in patients with serum TgAbs were high enough to diagnose LNMs [7].

**Table 4:** Review of diagnostic performances of FNA-Tg and the combination of FNA with FNA-Tg.

References	Cutoff values of FNA-Tg, ng/mL	Number of LNs, malignancy/benign	Sensitivity, %		Specificity, %		PPV, %		NPV, %		Accuracy, %	
			FNA-Tg	FNA with FNA-Tg	FNA-Tg	FNA with FNA-Tg	FNA-Tg	FNA with FNA-Tg	FNA-Tg	FNA with FNA-Tg	FNA-Tg	FNA with FNA-Tg
Snozek et al. 2007 [8]^a^	1.0	70/52	100	NA	96.2	NA	97.2	NA	100	NA	NA	NA
Kim et al. 2009 [12]^b^	10.0	119/49	90.8	94.1	89.8	87.8	95.6	95.7	80.0	86.0	90.5	92.3
Moon et al. 2013 [14]^b^	1.0	190/338	93.2	98.4	95.9	94.4	92.7	90.8	96.1	99.1	NA	NA
Jung et al. 2013 [15]^b^	1.8	77/100	96.1	100	94.0	93.0	92.5	91.7	96.9	100	94.9	96.1
Jeon et al. 2009 [18]^b^	36.0	56/20	94.6	96.4	90	90	96.3	96.4	85.7	90	92.3	93.2
Boi et al. 2006 [7]^b^	1.7 (absence of thyroid gland) / 36 (presence of thyroid gland) (sTgAb^-^)	19/32	100	NA	100	NA	NA	NA	NA	NA	NA	NA
	1.7 (absence of thyroid gland) / 36 (presence of thyroid gland) (sTgAb^+^)	14/8	100	NA	100	NA	NA	NA	NA	NA	NA	NA
Jeon et al. 2013 [22]^c^	10.0 (sTgAb^-^)	94/143	96.8	NA	95.9	NA	93.8	NA	97.9	NA	96.2	NA
	10.0 (sTgAb^+^)	13/12	69.2	NA	91.7	NA	90.0	NA	73.3	NA	80.0	NA
This study^c^	19.9 (sTgAb^-^)	90/117	91.1	96.7	100	100	100	100	93.6	93.6	96.1	96.1
	38.3 (sTgAb^+^)	16/16	75.0	100	93.8	93.8	92.3	94.1	79.0	100	83.3	96.9

^a^Included patients who previously underwent near-total or total thyroidectomy.

^b^Included all patients who did and did not undergo surgery for thyroid cancer.

^c^Included patients who underwent previous total thyroidectomy and radioactive iodine therapy.

FNA, fine needle aspiration; FNA-Tg, thyroglobulin level in FNA washout fluid; LN, lymph nodes; PPV, positive predictive value; NPV, negative predictive value; NA, not applicable; sTgAb-, undetectable serum TgAbs; sTgAb+, detectable serum TgAbs.

Contrary to the conclusions reached in these previous studies, there have been two pieces of evidence supporting that serum TgAbs actually affect the diagnositc performances of FNA-Tg to detect LNMs in patients with thyroid cancer [21, 22]. Cappelli et al. reported two cases in which the detection of FNA-Tg switched from undetectable to detectable levels in metastatic LNs after recombinant human thyrotropin stimulation in the presence of high levels of serum TgAbs [21]. Because serum TgAbs could fully prevent the detection of FNA-Tg in LNMs by saturating all the Tgs in the lymph nodes, they hypothesized that FNA-Tgs increased after thyrotropin stimulation, resulting in detectable levels in FNA washout fluid, even with the same titer of serum TgAbs [21]. Recently, Jeon et al. demonstrated a significant negative correlation between serum TgAbs and FNA-Tg levels, suggesting the possibility of interference by serum TgAbs [22]. On Tg stains of 4 malignant LNs from patients with serum TgAbs, the tissues were positive for Tg immunoreactivity although the FNA-Tg values were 10 ng/mL or less [22]. However, Jeon et al. still supported the clinical necessity of FNA-Tg tests in patients with serum TgAbs although they did not analyze the additional values of FNA-Tg to FNA according to the presence of serum TgAbs [22].

In this study, we investigated whether serum TgAbs might affect the detection of FNA-Tgs in diagnosing LNMs in patients who had undergone thyroidectomy and radioactive iodine ablation. Serum TgAbs significantly interacted with FNA-Tg measurements for LNM diagnosis on logistic regression analyses. The serum TgAbs modified the association between FNA-Tgs and LNMs, by decreasing ORs from 2.685–2.780 in the sTgAb^-^ group to 1.707–1.779 in the sTgAb^+^ group. We obtained different cutoff values of FNA-Tgs according to the presence of serum TgAbs, such as 19.9 ng/mL in the sTgAb^-^ group and 38.3 ng/mL in the sTgAb^+^ group. This higher cutoff value of FNA-Tg (38.3 ng/mL) in the sTgAb^+^ group could be explained by sTgAb interference, which would lead to an increased cutoff value of FNA-Tg for diagnosing metastatic lymph nodes. It means that sTgAb would not completely interfere with higher FNA-Tg levels. We also compared the diagnostic performances of FNA-Tg according to the absence or presence of serum TgAbs using two different optimal cutoff values of FNA-. The diagnostic performances of FNA-Tg were then compared according to the presence of serum TgAbs (Table 2). In the sTgAb^+^ group using a cutoff value of 38.3 ng/mL, all of the diagnostic values were decreased than those of the sTgAb^-^ group with a cutoff value of 19.9 ng/mL. We also compared diagnostic performances between FNA and the combination of FNA with FNA-Tg in the sTgAb^-^ and sTgAb^+^ groups, because of the interference of serum TgAbs in FNA-Tg measurements and decreased diagnostic performances of FNA-Tg in the sTgAb^+^ group (Table 3) [7, 10, 13, 22]. In the sTgAb^-^ group, sensitivity, NPV, accuracy and AUC were all significantly increased in the combination of FNA with FNA-Tg group, compared to the FNA only group. However, in the sTgAb^+^ group, the diagnostic performances of the combination of FNA-Tg to FNA showed decreased specificity and PPV than FNA alone, even though it showed superior sensitivity and NPV without statistical significance. Therefore, serum TgAbs likely interferes with FNA-Tg analysis and a negative result on FNA-Tg should be cautiously evaluated especially when the serum levels of TbAbs rise. And also the evaluation of TgAbs levels should be considered before performing FNA-Tg studies and further studies are needed to define appropriate critical threshold levels of FNA-Tg according to the TgAbs levels.

There were a few limitations in this study. First, this study was a retrospective study, and we included patients who underwent FNA, additional Tg measurements in FNA washouts, and lymph node dissections or imaging follow-ups for more than 1 year. Therefore, a selection bias could exist. Second, the small sample size of the sTgAb^+^ group (32 of 239 LNs) could reduce the statistical power of this study. Further studies with more patients will be needed to validate the diagnostic value of additional FNA-Tg measurements in the presence of serum TgAbs to diagnose LNMs. Third, a large proportion of benign LNs (133 of 239 LNs) were considered negative for LNMs based on imaging surveillance alone without surgical confirmation, which leaves the possibility for malignant LNs in some cases. And we suggested LNs with atypical cells on cytology, as a positive result even without final pathologic confirmation of malignancy according to the clinical and radiological evidence. The last limitation to be discussed is the cutoff value of 60 IU/L applied for serum TgAbs. There is debate that the cutoff value might be too high to detect the interfering effect of serum TgAbs. A previous study which used the same cutoff value also mentioned that any detectable level of serum TgAbs could influence the detection of Tg relevant to its level, and another study mentioned that the proof of interfering effect of TgAbs might be depend on cutoff value and methods because the reference ranges were not established yet [24, 29]. However, the value of 60 IU/L was the manufacturer-recommended cutoff value, and we applied the same cutoff value as recommended and as applied in a previous study. Further studies are needed to validate the true impact of serum TgAbs on the detection of FNA-Tg according to its level and different cutoff values.

In conclusion, serum TgAbs may interfere with FNA-Tg studies and caution is advised, but more work is needed to understand its true impact on management.

## Supporting Information

S1 Material Detail information of the patients including their FNA-Tg and TgAb levels. (DOCX)
