# Supplementary material for: A Study on Serum Antithyroglobulin Antibodies Interference in Thyroglobulin Measurement in Fine-Needle Aspiration for Diagnosing Lymph Node Metastasis in Postoperative Patients
Source: PLoS One. 2015 Jun 29;10(6):e0131096. doi: 10.1371/journal.pone.0131096 (PMC4488110; doi:10.1371/journal.pone.0131096)
Supplement: S1 Material — (DOCX) [file pone.0131096.s001.docx]

| **Standard Reference** | **FNA-Tg** | **Age** | **Gender** | **Size** | **Serum Tg** | **Initial cancer size** | **Extrathyroid extension** | **Multiplicity** | **Lymph node metastasis** | **TgAb** |
| --- | --- | --- | --- | --- | --- | --- | --- | --- | --- | --- |
| 1 | 0.2 | 52 | 0 | 6 | 0.20 | 7 | 1 | 0 | 1 | 27.8 |
| 0 | 0.2 | 59 | 0 | 14 | 0.22 | 5 | 0 | 0 | 0 | 21.51 |
| 0 | 0.2 | 69 | 0 | 4 | 0.20 | 10 | 1 | 0 | 0 | 11.04 |
| 1 | 0.2 | 68 | 0 | 10 | 0.20 | 14 | 0 | 0 | 0 | 9.9 |
| 0 | 0.2 | 61 | 0 | 9 | 1.33 | 25 | 1 | 1 | 1 | 7.67 |
| 0 | 0.2 | 54 | 0 | 8 | 0.20 |  |  |  |  | 5.5 |
| 1 | 0.2 | 29 | 0 | 9 | 0.20 | 15 | 0 | 1 | 1 | 5.5 |
| 0 | 0.2 | 56 | 1 | 11 | 0.20 | 27 | 0 | 1 | 1 | 23.4 |
| 0 | 0.2 | 66 | 0 | 10 | 0.20 | 5 | 0 | 0 | 0 | 21.2 |
| 0 | 0.2 | 40 | 0 | 5 | 0.20 | 25 | 1 | 0 | 1 | 5.5 |
| 0 | 0.2 | 52 | 0 | 12 | 0.20 |  |  |  |  | 10.24 |
| 0 | 0.2 | 46 | 0 | 12 | 0.20 | 15 | 1 | 1 | 1 | 10.87 |
| 1 | 0.2 | 30 | 0 | 6 | 0.20 |  |  |  |  | 5.5 |
| 1 | 0.2 | 69 | 0 | 22 | 0.58 | 30 | 1 | 0 | 1 | 46.15 |
| 1 | 0.33 | 45 | 0 | 17 | 0.2 | 6 | 1 | 1 | 0 | 5.5 |
| 1 | 0.38 | 68 | 0 | 20 | 0.20 |  |  |  |  | 9.9 |
| 1 | 0.84 | 29 | 0 | 8 | 0.20 | 20 | 0 | 0 | 1 | 9.2 |
| 0 | 0.2 | 63 | 0 | 8 | 0.20 | 23 | 1 | 0 | 1 | 20.4 |
| 1 | 19.9 | 39 | 0 | 5 | 0.20 | 14 | 1 | 0 | 1 | 54.49 |
| 1 | 32.9 | 41 | 0 | 10 | 0.20 | 5 | 0 | 0 | 1 | 21.12 |
| 1 | 35 | 61 | 0 | 7 | 0.26 | 21 | 1 | 0 | 1 | 27.83 |
| 0 | 0.2 | 55 | 0 | 10 | 0.20 | 8 | 1 | 1 | 0 | 20.3 |

**Supporting Information**

**S1 material.** Detail information of the patients including their FNA-Tg and TgAb levels.

FNA-Tg, thyroglobulin level in FNA washout fluid; Tg, thyroglobulin; TgAb, anti-thyroglobulin antibody
